# Providing Solution in an Emergency: COVID-19 and Voice Behavior of Healthcare Professionals

**DOI:** 10.1177/21582440221141700

**Published:** 2022-12-10

**Authors:** Muhammad Nawaz, Ghulam Abid, Talat Islam, Jinsoo Hwang, Zohra Lassi

**Affiliations:** 1 National College of Business Administration & Economics, Lahore, Pakistan; 2 Kinnaird College for Women, Lahore, Pakistan; 3 University of the Punjab, Lahore, Pakistan; 4 Sejong University, Gwangjin-gu, Republic of Korea; 5 The University of Adelaide, SA, Australia

**Keywords:** idiosyncratic deals (I-deals), display aggression, deontic justice, voice behavior, COVID-19

## Abstract

This study investigates the mechanism between idiosyncratic deals (I-deals) and voice behavior, considering display aggression and deontic justice as mediating variables. We collected data from 702 nurses and their immediate supervisors who work with COVID-19 patients through survey questionnaires at two different times, and we analyzed the data using structural equation modeling (SEM). We found that I-deals are significantly associated with deontic justice and voice behavior. Moreover, I-deals are significant but negatively associated with displayed aggression, which is significant and negatively associated with voice behavior. In addition, deontic justice and display aggression mediate the association between I-deals and voice behavior. These findings suggest that the hospitals’ top management should provide I-deals to nurses to improve their voice behavior.

## Introduction

COVID-19 has become the greatest challenge of all time for healthcare professionals across the globe (Ashfaq & Abid, 2022; Bilancini et al., 2020). This virus has infected 216 countries across the globe within a few months (Anser et al., 2020; Contreras, Baykal, & Abid, 2020), slowing down the global route to sustainability (Contreras, Abid, et al., 2020). The literature identified that COVID-19 emerged from a zoonotic virus, which is closely related to middle east respiratory syndrome (MERS) and severe acute respiratory syndrome (SARS) (Tang et al., 2020). On January 7, 2020, China officially declared the occurrence of this novel virus, which was named “severe acute respiratory syndrome coronavirus 2” (SAR-CoV-2), which included the understanding that it may lead to pneumonia (She et al., 2020). It was called COVID-19 afterward. According to World Health Organization (WHO), on October 10, 2022, 618,521,620 confirmed cases and 6,534,725 confirmed casualties had been reported, while as of October 4, 2022, a total of 12,723,216,322 vaccine doses have been administered globally (WHO, 2022b). The situation is not different in Pakistan because there were 1,572,972 confirmed cases with 30,623 confirmed deaths that have been reported, while as of September 25, 2022, a total of 310,629,295 vaccine doses have been administered (WHO, 2022c).

Healthcare service is a fundamental human right, and the state is responsible for providing healthcare facilities to its citizens. However, after the 18th Amendment to the Constitution, Pakistan transferred this responsibility to the provincial governments in 2010 (Javed & Liu, 2018). Pakistan is already facing a double burden of diseases, which include communicable and non-communicable diseases (CNCDs) that are still highly prevalent (Hydari et al., 2021; Pakistan Diseases Evaluation, 2017; Rana et al., 2021). The challenges that healthcare workers face are substantially greater than those encountered in their routine work, such as healthcare professionals are at risk while dealing with infectious diseases (Cox, 2020). Most healthcare professionals in the West also face healthcare challenges, such as engaging with patients with positive symptoms, connecting services, a population focus, providing services closer to home, and team care, which are common in other countries across the Asia-Pacific (Gauld, 2016). Unfortunately, the number of dissatisfied patients who receive healthcare services in Pakistani hospitals has continuously increased (Nawaz, Abid, et al., 2021). More specifically, around 70% of the population in Pakistan is serviced by private hospitals, whereas barely 30% is serviced by public sector hospitals. Therefore, the out-of-pocket healthcare expenses are 66.5%, whereas the world’s average is about 18% (Javed et al., 2019). According to the United Nations, Pakistan ranked 152nd out of 189th countries on the Human Development Index (The Nation, 2019), which 73% of the population does not fully recover in healthcare facilities, so they, therefore, have to undergo treatments on their own even after leaving hospitals (Hassan et al., 2017). Indeed, Pakistan has been spending less than 1% of its gross domestic product (GDP) on healthcare for over a decade (Pakistan Today, 2019), and healthcare is, therefore, not a priority in the country (Javed & Ilyas, 2018).

In the domain of health services, in developed and emerging economies, telehealth is an important measure to promote low-cost services (Fan et al., 2020). However, because of the weak information technology infrastructure in the Pakistani healthcare sector, even these new promising technologies cannot manifest their full strengths to lessen the burden on the country’s healthcare professionals and systems. Funds are not the only constraint because it is well-known that some developing countries, such as Cuba, have successfully managed to build and sustain a world-class healthcare system despite all their financial constraints (Javed et al., 2019). It can be argued that the country’s healthcare system is under immense pressure due to poor management, weak policies, and the inability to learn from the best practices abroad based on these facts. Indeed, an extension of communicable diseases, including COVID-19, disturbed the healthcare systems worldwide, particularly Pakistan’s healthcare system. A critical level that we need to understand this challenge is in terms of human behavior, particularly people’s propensity to engage in behaviors that are likely to slow the spread of the virus (Capraro & Barcelo, 2020; Everett et al., 2020; Van Bavel et al., 2020). Behavioral change is likely to hinge on people’s concerns and attitudes about the impacts of COVID-19 on their health, but subjective concerns could manifest at different levels.

From the behavioral perspective, the voice of the healthcare professionals is considered one of the main pillars to minimize the adverse effects and cope the healthcare issues to an extent. The key staff in the healthcare sector includes the nurses and doctors who directly deal with COVID-19 patients. Healthcare professionals’ guidance, attitude, and behavior matter a lot to prevent the spread of coronavirus during the current pandemic. A change in behavior, such as wearing face masks, is required from the general public to slow down COVID-19 transmission (Capraro & Barcelo, 2020). Similarly, we are arguing that the change in healthcare professionals’ behavior can also help to cope with the intensity of COVID-19 transmission. Van Dyne and LePine (1998, p. 109) defined voice behavior as a “promotive behavior that emphasizes the expression of constructive challenge intended to improve rather than merely criticize,” which is in line with the assessment mentioned above. Many healthcare workers choose not to raise their concerns (Tangirala & Ramanujam, 2008). Failure to speak up leads to missed opportunities for the organizational leaders to act upon and improve the work systems (Ilyas et al., 2021; Nawaz, Javed, et al., 2021; Nisar et al., 2020; Nisar et al., 2020; Yousaf et al., 2019).

Further investigation of the frontline health worker’s concerns can lead to resolutions and preventive measures to create safer and more efficient operational processes. Therefore, it is paramount for healthcare organizations to empirically test and measure the mechanisms that promote voice behavior among registered nurses in acute care settings, especially during the current pandemic. Therefore, coping with the recent pandemic to the extent in Pakistan and determining the importance of the voice behavior of healthcare professionals are the main focuses of this study. To accomplish this goal, exploring the antecedents of the voice behavior of the nurses is essential.

In this regard, the idiosyncratic deals (I-deals) of healthcare professionals have captured the attention of academicians and practitioners to cope with the current pandemic. It refers to the “personalized changes in work and employment conditions that individual workers have successfully negotiated with their employer or its agents” (Rousseau, 2005). The COVID-19 pandemic may be one of the greatest modern societal challenges requiring collective action and cooperation (Heffner et al., 2021). The shelter-in-place rule is also being asked to be adhered to by billions of people around the world to slow down the spread of the virus (Bilancini et al., 2020). Along with the medical grounds, the behavior of healthcare professionals also need to be examined to cope with the COVID-19 pandemic. Healthcare professionals with good behavior should be retained. By extending the arguments, the I-deals illustrate that idiosyncratic employment arrangements can be created to fit better the employees’ preferences, skills, and interests to motivate, attract, and retain talent (Liao et al., 2016). Another positive outcome of the I-deals could be that the recipient of I-deals are less likely to display aggression toward coworkers. Displaying aggression is an outcome of negative attributes, such as stress (Bodenmann et al., 2010), and it is also more likely that a recipient of the I-deals would treat other coworkers fairly, which involves deontic justice.

**Figure 1.**
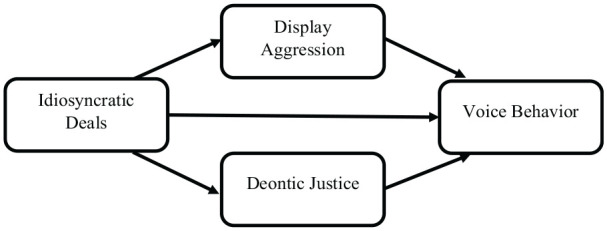
Conceptual model.

We claim that when these employment arrangements and personalized changes are settled with the healthcare professionals, they may feel happy and treat other individuals fairly, which is deontic justice. However, when an individual feels happy about their overall job attributes, they might not display aggression toward others while voicing their thoughts for the benefit of patients and hospitals. For instance, when healthcare professionals successfully negotiate their job tasks, careers, and work schedules, it is then more probable that they would feel happy, which also includes coping with stress while serving COVID-19 patients even with a minimum amount of equipment, which includes safety kits and medicine (Munawar & Choudhry, 2021). As a result, our study proposed that healthcare professionals should be provided the I-deals according to their preferences and interests to acquire their voices. In reaction to the initiating action, which includes the I-deals, the employees may choose to reciprocate this treatment with good or bad behavior of their own (Eisenberger et al., 1987), so we refer to these reactions as reciprocating responses. The social exchange theory (SET) posits that the targets will tend to respond with more positive reciprocating responses and/or fewer negative reciprocating responses in response to positive initiating actions. Therefore, the proposed model, illustrated in Figure 1, is supported by the social exchange theory (SET).

In general, a few steps, such as wearing face masks, social distancing, increasing hand washing, using disinfectants, and reducing face touching, have been determined to be necessary measures that can slow down the spread of COVID-19 (Van Bavel et al., 2020). The pandemic requires placing significant psychological burdens on individuals. The large-scale behavior changes reveal that with the recommendations of public health experts and epidemiologists, behavioral and social sciences can be used to help align human behavior with this crisis. Thus, our study might help improve the working environment in Pakistani hospitals during the COVID-19 pandemic to cope with these problems. Providing I-deals to healthcare professionals might help tackle these issues because these provisions can promote deontic justice and voice behavior of healthcare professionals by minimize the intensity of display aggression.

## Literature Review

### Idiosyncratic Deals and Voice Behavior

A voice is the employee’s response to ideas, opinions, and suggestions about job-related issues to improve the overall organizational processes (Abid et al., 2022). More precisely, a voice is defined as a “behavior that emphasizes the expression of constructive challenges to improve rather than merely criticize” (Van Dyne & LePine, 1998, p. 109). Conversely, to voice behavior, I-deals are categorized into tasks, careers, and flexible I-deals (Hornung et al., 2014), where the task I-deals are the personalized arrangements where an employee negotiates to make their job contents more rewarding and personally motivating (Hornung, Rousseau, et al., 2010). On the other hand, career I-deals refer to customized arrangements for securing professional advancement (Rousseau, 2005). These I-deals narrate proactive behavior related to skills development and career planning (Hornung et al., 2014; Seibert et al., 2001). The career I-deals are distinct from the task I-deals because they involve gaining motivation for tasks that are more strategically directed at improving employability (Grant, 2007; Parker & Collins, 2010). The career I-deals are more developmental and future-oriented than the task I-deals (Hornung et al., 2014). Therefore, it is most probable that the individuals that have these arrangements demand more job complexity, and the COVID-19 pandemic is an issue that requires more job complexity. Hence, career I-deals may provide more impulse to tackle the crises due to the extent of the issue. The third dimension of the I-deals involves the flexible I-deals, which involves personalized scheduling and work hours to fit better the individuals’ preferences and needs (Hornung, Glaser, & Rouseau, 2010). Flexible I-deals provide ways to condense the threats to an employee’s psychological wellbeing and the work overload of the staff by better aligning the fluctuations of the individual preferences with the work hours, which include the desired shifts, the work situations, and non-work activities (Kattenbach et al., 2010).

I-deals resemble other employment-related constructs, such as psychological contracts, job crafting, and negotiations, but they are distinct in nature (Liao et al., 2016). I-deals are observed in the literature as positively related to affective commitment, work engagement, and job satisfaction (Hornung, Glaser, & Rouseau, 2010; Hornung, Rousseau, et al., 2010; Rosen et al., 2013). Numerous studies provided the association of the I-deals with organizational outcomes, such as enhancing the level of job satisfaction of the I-dealers (Rosen et al., 2013), improving innovative work behavior (Kimwolo & Cheruiyot, 2019), strengthening the coworkers’ voice behavior (Marescaux et al., 2019), improving the organizational performance and the OCB (Dhiman & Katou, 2019), alleviating the work-family conflicts (Erden Bayazit & Bayazit, 2019), minimizing the organizational cynicisms (Morf et al., 2019), improving employee creativity (Wang et al., 2018), minimizing emotional exhaustion and deviant work behavior (Kong et al., 2015). However, the I-deals could be as effective during the COVID-19 pandemic theoretically and practically to the best of our knowledge.

According to Blau (1964), the SET suggests that individuals tend to reciprocate favors and contributions with partners in relationships. In the context of I-deals, the recipients feel obliged, so they opt to show positive work attitudes and behaviors that are in the employer’s favor. The SET supports this phenomenon in relation to the I-deals and voice. The employees’ reciprocation differs depending on their negotiation partner, which aligns with the assumption above (Lavelle et al., 2007), which could be the first-line manager, middle manager, or upper management. Ng and Feldman (2015) reported that the I-deals are significantly associated with voice behavior through the mediating role of organizational trust.

Similarly, Liu et al. (2013) found that with the assistance of the SET, the recipient of the I-deals is affectively committed and proactive, and they further recommended that exploring the association between the I-deals and voice behavior would be plausible. The discussion about whether I-deals promote voice behavior or not is still in the nascent stage, and little is known about the I-deals and voice behavior (Ng & Feldman, 2010). To address this association, Ng and Feldman (2015) sampled the United States and China’s professionals, and the same SET that was given by Blau (1964) was also considered in this study to address the relationship between the I-deals and the voice of the healthcare professionals. In social relationships, reciprocity is the prominent exchange (Ng & Feldman, 2015). Accordingly, we postulate that the recipients of the I-deals will reciprocate voice behavior. The I-deals are the employment arrangements where the supervisors are organized for positive outcomes as an exchange, and the I-deals of the healthcare professionals may lead to the voice behavior of nurses in favor of the hospitals in response. To the best of our knowledge, no study provides the association between the I-deals and voice behavior through the SET by sampling healthcare professionals, especially during the current pandemic. Consequently, based on the literature and the SET, it is most probable that the individuals within the system can demand a type of system or environment where they can voice their thoughts in the healthcare sector. As a result, the following hypothesis is postulated.

H1: I-deals positively effects voice behavior among nurses.

### Display Aggression as Mediator

Display aggression is defined as “negative acts that are perpetrated against an organization or its members and that victims are motivated to avoid” (Tedeschi & Norman, 1985, p. 30). As the variable is long-rooted, the existing literature reported its outcomes. For instance, displaying aggression lowers job engagement (Ford et al., 2016), job satisfaction, wellbeing, commitment (Ferris et al., 2016), life satisfaction, and self-esteem (Fida et al., 2018). Moreover, it enhances anxiety, turnover intentions (Ferris et al., 2016), stress (Malik et al., 2021), absenteeism, and burnout (Fida et al., 2018). These studies revealed that aggression produces negative consequences in the workplace, which still needs further investigation.

Initiating behaviors depends on the initial action of others (Nawaz et al., 2020). Organizational trust and support are considered positive initial actions at the workplace (Cropanzano & Rupp, 2008), whereas abusive supervision, workplace bullying, and incivility fall under the umbrella of negative initiating actions (Andersson & Pearson, 1999; Rayner & Keashly, 2005; Tepper et al., 2009). The SET explains that the recipients of the initiating action react according to the action received. For example, if the recipient perceives better tenets from the actor, she/he will respond positively. However, if the recipient perceives worse tenets from the actor, she/he will react negatively (Cropanzano et al., 2017).

Similarly, it is most probable on the bases of SET that if the recipients do not receive the I-deals, she/he might then react destructively. For instance, they may display aggression toward supervisors, coworkers, and patients. To extend this argument, the workload of dealing with COVID-19 healthcare professionals has dramatically increased during the current pandemic, whereas there is a shortage of nursing staff in Pakistan (Drennan & Ross, 2019), which requires providing I-deals to almost all the nurses if they require. Based on the exchange phenomenon, such as the SET, the feeling of happiness after receiving I-deals would be enhanced, so it would be most probable that the nurses would not display aggression toward their coworkers.

Therefore, keeping in mind these arguments, we can claim that employees, which are aggressive at the workplace, may not raise constructive voices. Also, employees facing aggression from their colleagues become victims of high psychological distress (Hershcovis & Barling, 2010). This psychological distress influences the motivational state of the individuals, which ultimately negatively influences the employees’ voice behavior. Based on the previous literature, employees who receive the I-deals would be happy with their job tasks and might not display aggression toward their coworkers, which may ultimately promote their voice behavior. As a result, we can claim that displaying aggression can mediate between I-deals and voice behavior. Thus, the following hypotheses are proposed based on the literature and the SET.

H2: I-deals negatively effects the display of aggression among nurses.
H3: Display aggression negatively affects voice behavior among nurses.
H4: Display aggression mediates the relationship between the I-deals and the voice behavior of nurses.

### Deontic Justice as Mediator

Folger (1998) developed Deontic justice as a construct, which refers to the extent to which actions and justice judgments derive from a sense of duty and moral obligations. Caring for oneself and others is the basic principle of deontic justice, which suggests that “a behavior is fair as long as it conforms to norms of moral obligation, not only for oneself but also for others” (Beugré, 2012, p. 2164). Even though this construct is long-rooted (Rupp & Bell, 2010), it cannot be measured and operationalized. However, the measurement and operationalization of deontic justice were required to get the individuals’ reactions. Therefore, the three dimensions of deontic justice, which include moral outrage, moral accountability, and moral obligation, were explored, explained, and verified by Beugré (2012). Moral outrage refers to the deontic response of resentment, anger, and indignation when an employee witness of injustice in the workplace (Beugré, 2012). Moreover, due to an implicit covenant within a human being, an individual has a moral obligation to act reasonably toward others, which an individual should not violate (Beugré, 2012). According to the denounce theory by Folger (2001), individuals try to govern interpersonal conduct on the grounds of moral accountability. People are most often held as the wrongdoers accountable for the perceived injustices. Consequently, “moral accountability serves as a line of defense against unfair conditions” (Folger et al., 2005).

The transgressors had to be punished for violating the human covenant so that the transgressors may treat others fairly. From the deontic perspective, this tendency is called the deontic effect, which portrays motivated cognition. For example, they feel a sense of moral unease when they witness others being mistreated. They should be motivated to address the injustice and react against the transgressors (Beugré, 2012; Rupp & Bell, 2010). Treating people fairly creates a sense of benefiting the organization, so it is most probable that the employees would voice their thoughts for the organization.

Although voice behavior has been discussed through the lens of the SET, its association with deontic justice and I-deals, illustrated in Figure 1, is missing with and without the support of SET. Recent meta-analyses on I-deals (Liao et al., 2016) demonstrate the growing need across countries, industries, and job groups to differentiate human resource practices. These practices in providing I-deals seem plausible to promote voice behavior in favor of the organization. Although I-deals’ benefits seem plausible from the recipients’ perspective, whether and how deontic justice could mediate between I-deals and voice behavior remains absent from the literature. A good literature review revealed that I-deals lead to voice behavior through the mediating role of flexible work role orientation, social networking behavior, and organizational trust (Ng & Feldman, 2015). However, the mediating role of deontic justice between I-deals and the voice behavior of healthcare professionals is still missing. We argue that based on SET, the recipient of I-deals would feel happy and, in return, treat coworkers fairly. An individual treated fairly, in return, may voice their thoughts in favor of the organization. Therefore, based on the dire need for the antecedents of voice behavior, the role of I-deals and deontic justice seems prominent, so our study is an attempt to fill this novel research gap.

Similarly, our study extends this argument by explaining the associations of the I-deals with deontic justice, deontic justice with voice behavior, and the mediating role of deontic justice between I-deals and voice behavior through the assistance of the SET during the current pandemic. In hospitals, when healthcare professionals negotiate their job tasks, careers, and work schedules according to their demands, it is more probable that they will treat other individuals fairly in response, including coworkers, patients, and supervisors. I-deals might be positively associated with deontic justice, and deontic justice can positively impact voice behavior. Consequently, based on the above arguments, deontic justice can probably mediate between the I-deals and voice behavior. As a result, we postulated the following hypotheses below.

H5: I-deals positively effects deontic justice among nurses.
H6: Deontic justice positively affects voice behavior among nurses.
H7: Deontic justice mediates the relationship between the I-deals and the voice behavior among nurses.

## Methods

### Sample and Procedure

We collected data from public and private hospitals in Pakistan using a questionnaire-based survey. The data was collected through dyads, which included nurses and doctors, to minimize the common method biases at two different times (Min et al., 2016). The data for the I-deals, display aggression, and deontic justice was collected from the nurses at Time 1 (T1), whereas the data about the voice behavior was collected from the immediate supervisors and the doctors of the same nurses at Time 2 (T2). The senior nurses are the supervisors of their subordinated nurses. However, since both the nurses and the head nurses work mainly under the supervision of the doctors in the operation theaters and other medical activities, the doctors are therefore considered the supervisors who can primarily evaluate the voice behavior of the nurses. Only the nurses working with COVID-19 patients were selected for the sample in this study. The university’s Internal Review Board (IRB) of the corresponding author approved this study. The ethical guidelines given by Fontana and Frey (2003) were followed during the data collection procedure. For example, the respondents were kept from mental and emotional interruptions during the data collection procedure.

We distributed 1,500 questionnaires at T1, and 837 nurses responded with a 55.8% response rate. At T2, their supervisors were approached, and only 702 responses were proper, which is an 83.87% response rate. The nurses were also asked about their demographic characteristics; most were female, representing 84.60% of the respondents. Moreover, 69.36% of the nurses were between 25 and 30 years old, held a diploma in the same field (74.89%), and 68.88% had more than 3 years of work experience.

### Measures

The idiosyncratic deals were measured using nine items that were developed by Hornung et al. (2014), which were evaluated on a 5-point Likert scale that ranged from (1) not at all to (5) to a very great extent. A sample item includes the job tasks that fit my strengths and talents.

Display aggression was assessed using the 10 items from Denson et al. (2006), which were evaluated using a 5-point Likert scale that ranged between (1) strongly disagree to (5) strongly agree. A sample item includes when someone or something makes her/him angry, she/he is likely to take it out on another person. The last four items of display aggression were deleted due to a loading issue, which is illustrated in the Appendix.

Deontic justice was measured using 18 items that were developed by Beugré (2012). These items were evaluated using a 5-point Likert scale that ranged from (1) never to (5) frequently. A sample item includes that I have a moral obligation to treat others fairly.

The voice behavior of the nurses was measured using the 10 items by Liang et al. (2012), which were evaluated using a 5-point Likert scale that ranged from (1) not at all satisfied to (5) satisfied to a very great extent. A sample item includes this nurse proactively developing and making suggestions for issues that may influence the unit.

### Analytical Strategy

We used the stem and leaf method to identify and manage the outliers. The Kaiser-Meyer-Olkin (KMO) technique with the maximum likelihood extraction method was used to investigate the factor loading of the items. The Cronbach’s Alpha (α) technique was used to examine the reliability of the variables. A confirmatory factor analysis (CFA) was also conducted using AMOS, which determined the maximum likelihood software for further validity. We followed Hair et al. (2010) suggestion for the model fit indices, which included the Chi-Square/Degree of Freedom (χ^2^/*df* ≤ 3.0), the Root Mean Square Residual (SRMR < .08), the Adjusted Goodness of Fit Index (AGFI ≥ .90), the Goodness of Fit Index (GFI ≥ 0.90), the Incremental Fit Index (IFI ≥ .90), the Tucker Lewis Index (TLI ≥ 0.90), the Comparative Fit Index (CFI ≥ .90), and the Root Mean Square Error of Approximation (RMSEA ≤ .08). The reliability of all the study variables was analyzed using Cronbach’s Alpha, which is the most widely applied index of internal consistency of the measure (Taber, 2018). The Cronbach’s Alpha values should equal to or exceed the minimum threshold of .70 (Nunnally & Bernstein, 1994). Furthermore, Pearson’s correlation coefficient (*r*) was used to inspect the direction and the strength of the bivariate relationships among the study variables. Finally, structural equation modeling (SEM) was used to test the study hypotheses.

## Results

We examined the data regarding the outliers, multicollinearity, and normality because these are the basic assumptions for applying structural equation modeling. The data for the study was collected face-to-face, and the respondents were requested to provide the missing values if the situation arose. As a result, there were no missing values in the data. We used the stem-and-leaf method for the outliers and found 12 responses with problematic values, so they were not included in the final analysis. Furthermore, skewness and kurtosis were found within the range of ±1 and ±3, and the correlation among the variables was less than .70, which shows the absence of multicollinearity (Tabachnick & Fidell, 2007). We also found that a single factor contributed to less than 50% of the variance. For a methodological measure, we tackled the issue of the common method bias by collecting data in dyads or from multiple sources, such as nurses and doctors (Podsakoff et al., 2003).

### Factor Loading and Discriminant Validity

The Kaiser-Meyer-Olkin (KMO) and Bartlett’s test with the maximum likelihood extraction method were used to investigate the factor loading of the items. It is common with the exploratory factor analysis (EFA) to use rule-of-thumb cutoffs to decide if an item/variable has a significant load on a respective factor with estimated standardized factor loadings of .30 to .40, which is often indicated as a meaningful and practically significant factor loading (Schmitt & Sass, 2011, p. 99). We found 0.914 as the value of KMO with the loading of items, which is shown in Appendix Table A. Furthermore, a novel approach for assessing the discriminant validity was introduced by Henseler et al. (2015), which is the heterotrait-monotrait ratio of correlations (HTMT). Moreover, the HTMT is relatively easy to compute. It only requires the correlations of the observed variables as the input. No exploratory or confirmatory factor analysis is needed. We found that the HTMT values are below 0.90, so there is no issue with discriminant validity (Henseler et al., 2015).

### Confirmatory Factor Analysis (CFA)

By following Hair et al. (2010), the values for Cronbach’s alpha (α ≥ .70), composite reliability (CR ≥ .60), and the average variance extracted (AVE ≥ .50) were examined. After fulfilling the basic assumptions, the CFA was conducted. The initial values of the model fit were not within the standard values (χ^2^/*df* = 4.56, CFI = .86, RMSEA = .89, and SRMR = .19) However, after deleting items with low factor loadings, which are illustrated in the measures, the values of the model fit improved (χ^2^/*df* = 2.97, CFI = .92, RMSEA = .067, and SRMR = .047).

### Descriptive Statistics

The values for the descriptive statistics are presented in Table 1. We noted that the respondents agreed on the I-deal (*M* = 3.55), deontic justice (*M* = 3.73), and voice behavior (*M* = 3.51). However, the mean score of display aggression (*M* = 2.97) shows that the respondents were neutral. We also noted a positive correlation of the I-deals with deontic justice (*r* = .47 and *p* < .01), voice behavior (*r* = .66 and *p* < .01), and a negative correlation with displayed aggression (*r* = −.37 and *p* < .01). Similarly, the correlation between displayed aggression and voice behavior was negative (*r* = −.38 and *p* < .01), but it was positive between deontic justice and voice behavior (*r* = .56 and *p* < .01). The values for Cronbach’s Alpha, AVE, and CR were also found to be within the used ranges, illustrated in Table 1.

**Table 1.** Reliability, Validity, Descriptive, and Correlation Analysis.

Variables	α	Mean	*SD*	AVE	CR	1	2	3	4
1-Idiosyncratic deal	.88	3.55	0.84	.53	.83	1			
2-Display aggression	.80	2.97	0.77	.58	.81	−.37**	1		
3-Deontic justice	.85	3.73	0.72	.61	.79	.47**	−.28**	1	
4-Voice behavior	.79	3.51	0.81	.59	.88	.66**	−.38**	.56**	1

*Note.* α ≥ .70.

** *p* < .01.

### Hypotheses Testing

We used structural equation modeling (SEM) to test the effects of the independent variable on the dependent variables, which is illustrated in Table 2. We noted the positive effects of the I-deal on voice behavior (β = .48 and *p* < .01) and deontic justice (β = .47 and *p* < .01), and the negative effect on displayed aggression (β = −.37 and *p* < .01). These values support H1, H5, and H3. Furthermore, we noted a negative effect of displayed aggression on voice behavior (β = −.12 and *p* < .01) and a positive effect of deontic justice on voice behavior (β = .31 and *p* < .01), which support H3 and H6.

**Table 2.** Hypotheses Testing Through Hierarchical Regression.

Relationships	β	CR	*SE*	*p*	Hypotheses
Idiosyncratic deal→voice behavior	.48	15.549	0.030	**	H1 is accepted
Idiosyncratic deal→display aggression	−.37	−10.474	0.039	**	H2 is accepted
Display aggression→voice behavior	−.12	−4.315	0.024	**	H3 is accepted
Idiosyncratic deal→deontic justice	.47	14.049	0.035	**	H5 is accepted
Deontic justice→voice behavior	.31	10.794	0.028	**	H6 is accepted

** *p* < .01.

We tested the mediating hypotheses through 5,000 bootstraps at the 95% confidence level and identified the upper and lower boundaries. Regarding the mediating role of display aggression between the I-deal and voice behavior, the indirect effect (β = .05, LL = 0.033, UL = 0.088, and *p* < .01) was found to be less compared to the direct effect (β = .48 and *p* < .01). However, there was no zero between its upper and lower limit, so H4 is accepted. Similarly, regarding the mediating role of deontic justice between the I-deal and voice behavior, the indirect effect (β = .17, LL = 0.113, UL = 0.199, and *p* < .01) was found to be less compared to the direct effect (β = .48 and *p* < .01). However, there was no zero between its upper and lower limit, shown in Table 3, so H7 is accepted.

**Table 3.** Mediation Analysis Using Bootstrap.

Relationships	β	*p*	Bootstraps @ 95%		Hypotheses
			LL	UL	
I-Deal→display aggression→voice behavior					
Direct effect	.48	**	0.403	0.543	H4 is accepted
Indirect effect	.05	**	0.033	0.088	
I-Deal→deontic justice→voice behavior					
Direct effect	.48	**	0.403	0.543	H7 is accepted
Indirect effect	.17	**	0.113	0.199	

** *p* < .01

## Discussion

Nurses’ often careless and rude behavior toward patients has been observed in the health sector (Becker et al., 2018), which has continued during the current pandemic (Babvey et al., 2020). This kind of attitude and behavior of nurses may be due to a number of reasons, such as a shortage of nursing staff, work overloads, hectic job timings, and personal risks, such as the fear of infections due to dealing with infectious diseases. Our study claims that these issues can be managed by providing I-deals to the nurses, and their voice behavior would also be augmented. Therefore, management support in the I-deals is required to manage debacle environments and get the best out of voice behavior. The consequence of providing I-deals to nurses would not limit to voice behavior but their level of aggression can be controlled and they would also treat each other fairly. Hence such a flourishing environment would be produced where healthcare professional can perform creatively.

People change their behavior toward preventive measures by adopting the shelter-in-place rule all over the globe. This behavioral change includes wearing face-covering masks, using disinfectants, and social distancing, which has to be ensured to minimize the spread of the COVID-19 disease. These behavioral interventions are mandatory for individuals living in a society and are also required by healthcare professionals. The voice behavior of healthcare professionals would be one of the behavioral interventions, so our study mainly focused on the voice behavior of healthcare professionals toward coping with the current COVID-19 pandemic. We found the significance given this fact, but a negative association between I-deals with display aggression is further significant and positively associated with voice behavior. Similarly, we found a significant and positive association of I-deals with deontic justice, which is significantly and positively associated with voice behavior. The present investigation has three major findings, which align with the assumption above. First, if the I-deals are nurtured with the requirements of the healthcare professionals, their voice behavior will significantly improve, and the unit operations used to cope with the COVID-19 disease may operate effectively. Second, the display of aggression of the healthcare professionals significantly mediates the association of the I-deals and voice behavior; such as, by providing the I-deals to the healthcare professionals, their display of aggression is minimized, and their voice behavior significantly improves. Third, the deontic justice of the healthcare professionals mediates the association of the I-deals and voice behavior; such as by providing the I-deals to the healthcare professionals, their sense of deontic justice improves, which significantly and positively influences the voice behavior.

**Figure 2.**
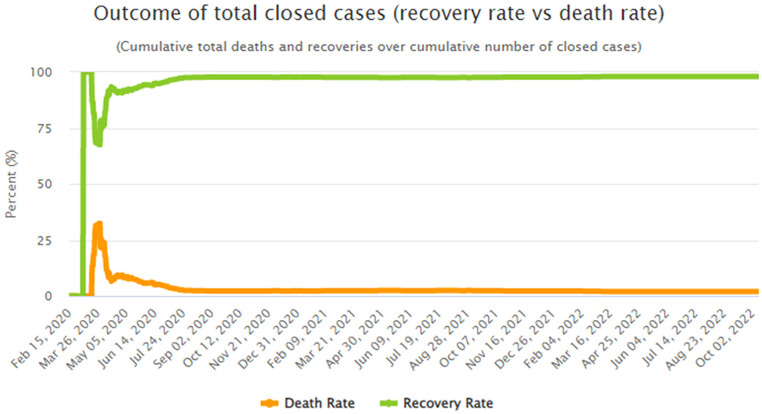
Recovery rate versus death rate. *Source*. https://www.worldometers.info/coronavirus/country/pakistan/, available online on October 10, 2022.

The voice of the front-line employees is always considered effective from the academic as well as from a practitioner’s point of view (Nawaz et al., 2018). Similarly, the nurses’ voice matter in improving the hospital’s quality of services (Islam et al., 2019). Considering this fact and to the best of our knowledge, since the voice of the nurses in terms of the I-deals, display aggression, deontic justice, and COVID-19 has not been captured to date, our findings have therefore been drowned by focusing on the voice of the nurses in this study. The findings recommend that the I-deals be provided to the nurses to improve their constructive voice, which would help in dealing with COVID-19 patients. Since the shortage of nurses is already a big issue in Pakistan (Karamat et al., 2019), therefore, instead of hiring a new workforce, which is a time-consuming process, hospitals should provide I-deals to the nurses to manage the shortage to activate both the government and the hospitals’ higher authorities. Besides the likely consequences of voice behavior, we found there are many confirmed cases, and the recoveries in Pakistan are also better compared to other countries, which include the USA, India, France, and Brazil, as of October 10, 2022. Although, the recovery rate of COVID-19 patients is high as compared to death rate, as illustrated in Figure 2, but the life of an individual citizen matters. Therefore, if the voice of healthcare professionals can safe even a single individual, then it should be nurtured and examined ethically during the current pandemic.

## Implications, Limitations, and Recommendations

### Implications

The study has several theoretical and practical implications for healthcare administrators and professionals, for instance, literature explains the nurses’ voice behavior (Islam et al., 2019). However, the role of the I-deals, display of aggression, and deontic justice to shape voice behavior have been neglected. Examining the proposed model, shown in Figure 1, in the current pandemic is imperative to study as an extension. This research adds to the debate about coping with the COVID-19 disease from a behavioral perspective by contributing to a growing body of knowledge about the implications of organizational behavior and human resource management. Specifically, the findings from this study revealed that the hospital management should focus on the I-deals of the healthcare professionals to gain fruitful consequences instead of focusing on their weaknesses. The current study found that the voice behavior of the nurses is limited in the presence of display aggression, but it is significantly improved in deontic justice. This highlights the role of the I-deals, which illustrates that if the hospital management provides it, the display of aggression could be minimized, and demotic justice could be maximized to obtain positive consequences, such as voice behavior. Furthermore, the current study contributed to the SET by providing the I-deals to the healthcare professionals in exchange for adverse outcomes, which include display aggression being minimized, and positive outcomes, which include deontic justice and voice behavior, that can be maximized regarding dealing and coping with the COVID-19 pandemic up to an extent.

This study has several practical implications for medical professionals, organizational behaviors, and human resource management. First, this study provides suggestions that depict how healthcare professionals make I-deals to raise concerns regarding the value of hospitals by highlighting the positive aspects of the I-deals. We suggest that the healthcare sector consider the I-deals as an integral part of the healthcare professionals handling unique challenges. The hospitals can take several steps to enhance the nurses’ voice behavior. This study focuses on providing job-related support through formal employment arrangements, improving the deontic response of the nurses, and minimizing the intensity of the display of aggression. Second, Mawritz et al. (2012) suggested that both the negative and positive behavior of the supervisors can trickle down to the subordinates’ behavior, so the training of doctors and supervisors from a behavioral point of view can enhance the motivation of the nurses to promote their voice behavior to deal with the COVID-19 pandemic effectively. Third, it is critical that the policymakers consider the nurses’ opinions because their opinions can provide hands-on management information about various COVID-19 patients. Thus, management should create an environment where healthcare professionals feel it is easy to provide their observed opinions. The policymakers should be as unbiased as possible with making decisions (Mustafa et al., 2022), which can improve the environment where the nurses can voice their thoughts in general and value the hospitals by effectively curing COVID-19 patients.

### Limitations and Future Directions

This study has several limitations as well as future directions. The diverse sample includes 702 nurses from a metropolitan city, which is feasible regarding demographics. However, we cannot claim that it represents Pakistan. Thus, the generalizability is limited, so future studies should focus on other cities and countries to test the same model to make this study more general. It can be tested in other countries because COVID-19 is a global pandemic (WHO, 2020a,b,c).

A limitation of the study includes the cross-sectional time-lagged nature of the data. It would be better if the given model were tested at the beginning of the COVID-19 pandemic, as study 1 and compared with a model tested during the current time, as study 2. A very direct consequence of the I-deals and voice behaviors could be observed. Therefore, future researchers should now try to test the model in another culture, as in Study 2, and create a discussion of the behavioral perspective by comparing it with this study. Therefore, the longitudinal and experimental design of the study can be nurtured in the future. Furthermore, the study involves female nurses, mainly focused on the doctors, who were just considered their supervisors. Even though the doctors are the primary workforce of the hospitals, their I-deals, which displayed aggression, deontic justice, and voice behavior, were neglected in this sense. This can ultimately influence the nurses’ attitudes and behaviors. Therefore, future studies should focus on the doctors’ voice behavior to make the study more rigorous and applicable.

The data was collected from private and public hospitals, but no comparisons were discussed, which limits the study. Thus, future studies can compare for better suggestions to cope with the COVID-19 pandemic from a behavioral perspective. The study variables can be influenced by the other moderating variables, such as stress, cyber loafing, self-efficacy, workloads, and workplace bullying, which are neglected and reveal the study’s limitations. Thus, future studies should focus on these variables to make the study more applicable and practical.

## Conclusion

This study examined the link between the I-deals, display aggression, deontic justice, and voice behavior of healthcare professionals to minimize and cope with the spread of the COVID-19 disease from a behavioral perspective. The results of this study revealed that based on the tenet of SET, the I-deals significantly improve the voice behavior and deontic justice of the nurses and minimize their level of display aggression, which influences the COVID-19 pandemic in terms of minimizing its intensity. Therefore, hospitals should devote greater attention to providing I-deals to the nurses to gain positive outcomes in dealing with the COVID-19 pandemic. We hope our study contributes to the literature on coping with the COVID-19 pandemic by discussing the new paradigm involving healthcare professionals’ behavior. The change in individuals’ behaviors, such as wearing face masks, keeping social distancing, and the use of disinfectants (Capraro & Barcelo, 2020; Everett et al., 2020; Heffner et al., 2021; Pfattheicher et al., 2020; Raihani & De-Wit, 2020) was discussed about the new paradigm that is mentioned above. However, the healthcare professionals’ behavior change needs to be examined, so our study suggests that voice behavior would be a positive parameter in this regard, and I-deals would be a pivotal element in augmenting the nurses’ voice.

## Appendix

**Appendix.** Factor Loadings.

Variable	Items	Factor loading
I-deals	Job tasks fit my personal strengths and talents.	0.464
	Job tasks fit my personal interests.	0.420
	Personally, motivating job tasks.	0.603
	Career options suit my personal goals.	0.645
	Personal career development opportunities.	0.579
	Ways to secure my professional advancement.	0.690
	A work schedule suited to me personally.	0.664
	Extra flexibility in starting and ending my work day.	0.614
	A work schedule customized to my personal needs.	0.541
Deontic justice	I have a moral obligation to treat others fairly.	0.662
	Treating others fairly should be a moral obligation for everyone.	0.750
	Treating people with respect and dignity is a moral obligation.	0.736
	I have a moral obligation to uphold the principles of fairness.	0.398
	It is important for me to see that others are treated fairly.	0.705
	For me, treating others fairly is a moral duty.	0.734
	Fairness is a moral virtue.	0.784
	I deeply care about fairness.	0.760
	People who treat others unfairly should be held accountable.	0.677
	It is important to hold people accountable for their failure to follow fairness rules.	0.522
	People should be confronted when they act unfairly.	0.562
	Violators of fairness should be held accountable.	0.634
	It is important to identify violators of fairness.	0.664
	Being held accountable should be a must when people act unfairly.	0.696
	I feel sad when I see others being unfairly treated.	0.653
	It bothers me when I see that others are not being fairly treated.	0.664
	I feel saddened by injustices done to others.	0.579
	I am concerned by unfairness done to others.	0.356
Display aggression	When someone or something makes me angry, I am likely to take it out on another person.	0.733
	When feeling bad, I take it out on others.	0.804
	Sometimes I get upset with a friend or family member even though that person is not the cause of my anger or frustration.	0.728
	I take my anger out on innocent others.	0.681
	When things don’t go the way I plan, I take out my frustration on the first person I see.	0.709
	If someone made me angry, I would likely vent my anger on another person.	0.632
Voice behavior	Proactively develops and make suggestions for issues that may influence the unit.	0.558
	This nurse proactively suggests new projects which are beneficial to the work unit.	0.703
	Raises suggestions to improve the unit’s working procedure.	0.529
	Proactively voices out constructive suggestions that help the unit reach its goals.	0.476
	Makes constructive suggestions to improve the unit’s operation.	0.311
	Advises other colleagues against undesirable behaviours that would hamper job performance.	0.330
	Speaks up honestly with problems that might cause serious loss to the work unit, even when/though dissenting opinions exist.	0.549
	Dares to voice out opinions on things that might affect efficiency in the work unit, even if that would embarrass others.	0.650
	Dares to point out problems when they appear in the unit, even if that would hamper relationships with other colleagues.	0.513
	Proactively reports coordination problems in the workplace to the management.	0.509
